# Notes and News

**Published:** 1900-12-22

**Authors:** 


### Notes and News.

In response to the appeal recently issued, the Prince of Wales's Hospital Fund has received £2,000 from Mr. Julius Wernher, £500 from Mr. Alfred Beit, and £100 from Mr. Lionel Phillips.

The Council of King's College, London, have elected Dr. Arthur Robinson, at present Lecturer on Anatomy in the Middlesex Hospital Medical School, to the Professorship of Anatomy, in succession to the late Professor Hughes.

Professor Clowes, Chemical Adviser to the County Council, delivered a lecture before the Society of Arts last week on the treatment of London sewage. The lecturer described the present method pursued, which he maintained was not wholly satisfactory, but he did not bring forward any alternative proposals. It was pointed out by a subsequent speaker that the water treatment was wasteful of fertilising elements which might be applied to the land.

The *Star*, commenting on our article entitled "The Scandal of 'Hospital Scandals,'" protesting against the habit which that journal has of heading any complaint against any of the hospitals as "Another Hospital Scandal," in its reply ignores our main point. That point was, that in the recent cases published by the *Star* under the objectionable heading referred to, the writer showed ignorance of hospital matters by advising the public not to subscribe to the voluntary hospitals so long as these so-called scandals continued. The hospitals attacked were St. Bartholomew's Hospital, to which the public contributes nothing as it is wholly endowed; and Guy's Hospital, another endowed institution, for which contributions from the public have only been asked during recent years. We welcome the general fairness of the *Star's* reply, and hope that in future they will not condemn the voluntary hospitals which are wholly dependent on public support on account of errors in management attributed by the *Star* to endowed institutions.

Lord Mountstephen has promised £25,000 to clear the Aberdeen Royal Infirmary from debt.

The late Mr. Henry Chester, of Putney, has made a will whereby £60,000 will at once be available to endow a hospital, should one be erected at Putney. This offer remains open for twenty years, and every year the interest on the money will accumulate to swell the sum available. If within twenty years no hospital is erected, Guy's Hospital is to benefit and receive the whole amount.

The Malaria Expedition sent out to West Africa by the Liverpool School of Tropical Medicine have published their conclusions, which are of a practical nature. They find that the only two methods of prevention in these districts which can be adopted are the segregation of Europeans from natives of all sorts, at a distance of about half a mile, and secondly the complete and efficient surface drainage of the whole district in the immediate neighbourhood of European quarters. The Commissioners report that in some cases these measures might be easily adopted at once, but would be costly and difficult in others, but that where new settlements are made they should be adhered to.

Complaints have been made that the privileges of the Aberdeen Royal Infirmary were taken advantage of by patients who were able to pay for treatment. The chairman, Mr. G. M. Cook, has replied that this is rarely the case, and that a scrutiny of the patients' books at the infirmary would convince those who complain that not only did not the well-to-do receive free treatment but that the poor were doing their best out of their poverty to contribute towards the expenses incurred for them. All patients at the Royal Infirmary are given an opportunity of contributing, and each year showed an increase in the amount received from this source.

A deputation from the County Councils' Association was received by the President of the Local Government Board at the House of Commons, petitioning that the Government should support or take up a Bill next session, to amend the Isolation Hospital Act of 1893, by giving County Councils power to acquire hospitals built and established under the Public Health Act of 1875, or to contribute to the maintenance of these hospitals. The petition is presented partly in view of the fact that in case of epidemic there were not at the present time a sufficient number of isolation hospitals. Mr. Long, in receiving the deputation, stated that he would himself draft a Bill and advocate its adoption.

Colonel Otter, Commander of the Royal Canadian Regiment, has written to thank the Colonial Troops Entertainment Committee for the arrangements made for their entertainment during the visit of the regiment to London. The men were highly delighted at the hospitality and kindness shown them. This is very satisfactory, and we can only hope that the Entertainment Committee will find means to secure equally successful hospitality for those other Colonial troops who will visit London on their way home. One City firm has already promised £1,000, and a further £500 is forthcoming from a private source. But more is needed, and this is a work to which all can contribute.

